# Predicting the Mosquito Species and Vertebrate Species Involved in the Theoretical Transmission of Rift Valley Fever Virus in the United States

**DOI:** 10.1371/journal.pntd.0003163

**Published:** 2014-09-11

**Authors:** Andrew J. Golnar, Michael J. Turell, A. Desiree LaBeaud, Rebekah C. Kading, Gabriel L. Hamer

**Affiliations:** 1 Department of Entomology, Texas A&M University, College Station, Texas, United States of America; 2 Virology Division, United States Army Medical Research Institute of Infectious Diseases, Fort Detrick, Frederick, Maryland, United States of America; 3 Center of Immunobiology and Vaccine Development, Children's Hospital Oakland Research Institute, Oakland, California, United States of America; 4 Division of Vector-Borne Diseases, Arbovirus Diseases Branch, Centers for Disease Control and Prevention, Fort Collins, Colorado, United States of America; University of California, Davis, United States of America

## Abstract

Rift Valley fever virus (RVFV) is a mosquito-borne virus in the family *Bunyaviridiae* that has spread throughout continental Africa to Madagascar and the Arabian Peninsula. The establishment of RVFV in North America would have serious consequences for human and animal health in addition to a significant economic impact on the livestock industry. Published and unpublished data on RVFV vector competence, vertebrate host competence, and mosquito feeding patterns from the United States were combined to quantitatively implicate mosquito vectors and vertebrate hosts that may be important to RVFV transmission in the United States. A viremia-vector competence relationship based on published mosquito transmission studies was used to calculate a vertebrate host competence index which was then combined with mosquito blood feeding patterns to approximate the vector and vertebrate amplification fraction, defined as the relative contribution of the mosquito or vertebrate host to pathogen transmission. Results implicate several *Aedes* spp. mosquitoes and vertebrates in the order Artiodactyla as important hosts for RVFV transmission in the U.S. Moreover, this study identifies critical gaps in knowledge which would be necessary to complete a comprehensive analysis identifying the different contributions of mosquitoes and vertebrates to potential RVFV transmission in the U.S. Future research should focus on (1) the dose-dependent relationship between viremic exposure and the subsequent infectiousness of key mosquito species, (2) evaluation of vertebrate host competence for RVFV among North American mammal species, with particular emphasis on the order Artiodactyla, and (3) identification of areas with a high risk for RVFV introduction so data on local vector and host populations can help generate geographically appropriate amplification fraction estimates.

## Author Summary

In anticipation of continued pathogen emergence in the U.S. due to globalization climate change, and other factors, the development of proactive management plans and interventions to predict and then intervene is going to be more efficient and effective than retrospective plans developed after pathogen emergence. Effective management of mosquito-borne pathogens like Rift Valley fever virus (RVFV) requires an understanding of the roles that different mosquito species and vertebrate hosts play in transmission. This study combines data on mosquito transmission efficiency, mosquito feeding patterns, and vertebrate infectiousness to quantitatively evaluate the relative importance of different mosquito species and vertebrate hosts to the amplification of RVFV in the U.S. We identify several species of floodwater *Aedes* spp. mosquitoes that would be the most likely vectors for RVFV, and hoofed ungulates (deer, cows, sheep) would be the most important amplifying vertebrate hosts. Although these data provide public and animal health agencies *a priori* knowledge on the primary mosquitoes that should be targeted for vector control and the highest priority animals to receive vaccines, this analysis reveals many gaps in knowledge reducing our ability to predict and then manage a potential invasion of RVFV.

## Introduction

Rift Valley fever virus (RVFV) is an emerging infectious disease in Africa and the Middle East. If introduced to North America, RVFV is capable of serious health and socioeconomic consequences potentially incapacitating large numbers of humans, decimating susceptible farm animals, and instigating heavy restrictions on livestock trade [1], [2]. Although transmission of the virus can occur through aerosol inhalation or direct tissue-tissue contact by handling of infected organisms, an enzootic cycle between mosquito vectors and domestic or wild animals has been repeatedly proposed as a main mechanism of transmission [3]. Clinical signs vary by vertebrate species and age, but infected pregnant ruminants generally suffer spontaneous abortions and juvenile ruminants suffer high mortality while occasional spillover into human populations results in a self-limiting, febrile illness that may progress to encephalitis, retinitis, blindness, hemorrhagic fever or death [2]–[5]. In 1931, RVFV was first reported in Kenya. It spread to Egypt in 1977 and was detected on the Arabian Peninsula in 2000 [6], [7]. Since advancing beyond African borders in 2000, total human cases of RVFV include 768 confirmed fatalities, 4,248 confirmed infections and over 75,000 suggested unconfirmed cases [8]–[15].

The emergence of arthropod-borne viruses (arboviruses) through geographic expansion is facilitated when amplification hosts include wild or domestic animals, as demonstrated by West Nile virus (WNV), Japanese encephalitis, and epizootic hemorrhagic disease [2], [16]. *Aedes* and *Culex* spp. mosquitoes are proposed to be the main vectors of RVFV, where *Aedes* spp. act as the reservoir and maintenance vectors that emerge after flood events and feed heavily on livestock [17]. *Culex* spp. mosquitoes then become involved as amplifying hosts of RVFV leading to epizootics and the eventual spillover to human populations [5], [17]–[19]. However, the understanding of RVFV transmission biology in Africa and the Arabian Peninsula remains underdeveloped. Additionally, unresolved questions surround endemic persistence of the virus, such as transovarial transmission [17].

Should RVFV arrive, diagnosing the disease and controlling the spread of infected vertebrates will take time, and proactive management plans should be created to minimize the time to react and break transmission of the pathogen. Even though RVFV is identified as an emerging infectious disease threat and is classified as a “Category A select agent” by both the Centers for Disease Control and Prevention and the US Department of Agriculture, gaps in data are preventing a proper evaluation of the different roles vectors and vertebrate hosts potentially may play in RVFV transmission in the U.S. beyond qualitative conjecture [1], [20]. To prepare for an arbovirus introduction, it is essential to understand which vectors and vertebrate hosts may be responsible for viral amplification and transmission, as disease control methods vary depending on the target species [21], [22]. For example, mosquito species using small container habitats for larval development are often controlled using larvicides and source reduction of aquatic habitat, whereas mosquito species with synchronous emergence following flooding events are controlled by adulticides or granular larvicides applied prior to flooding [23], [24].

To assess the role of mosquitoes and hosts in the transmission of a virus, it is important to quantify the ability for a mosquito species to transmit a pathogen (vector competence), the infectiousness of vertebrate host species (host competence), and contact rates between mosquitoes and vertebrate hosts. In the WNV system, Kilpatrick et al. [25] combined data on vector competence, abundance, and mosquito feeding patterns to identify the species of mosquitoes responsible for bridge transmission of WNV to humans. Several studies have then implicated important avian hosts disproportionately responsible for WNV amplification based on mosquito host feeding patterns, mosquito vector competence data, and vertebrate host competence data [26], [27]. By applying models utilized in the WNV system, we can implicate potentially important vectors and vertebrate hosts in RVFV transmission should the virus arrive. A number of reviews discuss potential vertebrate hosts, disease vectors, and environments that may support RVFV transmission in the U.S., through environmental receptivity models [28] and spatial overlap of important host populations [22]. However, to our knowledge, no study has quantitatively evaluated the theoretical importance of different mosquito species and vertebrate hosts to RVFV transmission and amplification in the U.S. [28].

This study utilized published and unpublished vector and host competence data and mosquito feeding patterns to model the theoretical roles of different mosquito and vertebrate species in the amplification and transmission of RVFV in the U.S. Although predictions from this analysis are strictly theoretical, and limited by available data, these results highlight critical gaps in knowledge necessary to properly evaluate the potential transmission activity of RVFV in the U.S. and provide hypotheses that can support proactive arbovirus surveillance and control programs.

## Methods

### Vector competence

Mosquito vector competence studies evaluate the ability of mosquitoes to develop an infection and ultimately transmit the pathogen during feeding. Data generated from vector competence studies include viral dissemination and transmission rates. Viral dissemination rates are defined as the percentage of orally exposed mosquitoes with virus detected in their legs seven or more days after RVFV infection. Transmission rates are defined as the percentage of orally exposed mosquitoes (regardless of infection status) that transmitted virus by bite upon refeeding [21]. Selected studies evaluated mosquito species that occur in the U.S. and monitored dissemination and transmission rates after feeding on a RVFV infected animal at the incubation temperature of 26°C. RVFV vector competence studies were located using Web of Science, NCBI's Pubmed, and the Armed Forces Pest Management Board Literature Retrieval Systems [21], [29]–[35].

Analyzing viral dissemination and transmission data drawn from multiple studies is problematic because these data are dependent on the viremic titer of exposure [33] and the compiled transmission data for this analysis reflects mosquitoes exposed to viremia that ranged from 10^4.3^ to 10^10.2^ plaque-forming units/ml (PFU/ml). To address this issue, a regression analysis of log viremia versus experimental transmission data from 17 mosquito species (Figure S1, A and B) was utilized to estimate the dependence of dissemination and transmission rates on viremic dose. Slopes from these regressions were combined with experimental data from each mosquito species to interpolate what the dissemination and transmission rates would be at the exposure viremia of 10^7.5^ PFU/ml (equations shown in Table S1). Mosquito species that demonstrated low overall vector competence in experimental transmission studies due to midgut escape barriers or salivary gland barriers (i.e. *Anopheles crucians* (Wiedemann), *Cx. nigripalpus* (Theobald) and *Ae. infirmatus* (Dyar & Knob)) or had a limited sample size (N<2 mosquitoes) were not used in the regression analyses [29].

The viremia-dissemination equation was equal to 0.098*(Log_10_ viremia) −0.268 and the viremia-transmission rate of a mosquito with a disseminated infection equation was equal to 0.056*(Log_10_ viremia)−0.0155 (Figure S1, A and B; Table S1). Both equations show a positive relationship for dissemination (N = 27; R^2^ = 0.28; p = 0.0049) and transmission (N = 27; R^2^ = 0.13; p = 0.07) as viremic dose increases. For each mosquito species we generated a linear equation and the y-intercept was adjusted for each mosquito species based on the difference between the experimentally observed rate and what the standardized equations described above (Figure S1, A and B) would predict at a specific viremic dose. This adjusted y-intercept and the standardized slopes from Figure S1, A and B (Dissemination m = 0.098, Transmission m = 0.056) were utilized to create two unique linear equations for each mosquito species: one to calculate dissemination rate and one to calculate transmission rate with respect to viremic dose for each vector species. By solving for y when x = log_10_ 7.5 PFU/ml we were able to estimate dissemination and transmission rates at an exposure viremia of 10^7.5^ PFU/ml for each mosquito species (Table 1, Table S1). When there were multiple data points for a mosquito species the averages of exposure viremia and the observed experimental transmission data were used to calculate the two linear equations for vector competence standardization.

**Table 1:** Estimated dissemination rate, transmission rate, and vector competence for mosquitoes exposed to 7.5 log PFU/ml Rift Valley fever virus.

Species [citation]	Dissemination rate^a^	Transmission rate^b^	Vector Competence (Cv)^c^
*Coquillettidia perturbans* [29]	0.53	0.72	0.38
*Aedes j. japonicus* [30]	0.74	0.51	0.37
*Culex tarsalis* [31], [32]	0.38	0.87	0.33
*Aedes excrucians* [31]	0.28	1.00	0.28
*Aedes canadensis* [31]	0.70	0.40	0.28
*Aedes sollicitans* [31]	0.76	0.34	0.25
*Aedes triseriatus* [31]	0.75	0.32	0.24
*Psorophora ferox* [29]	0.55	0.32	0.18
*Culex territans* [31]	0.39	0.45	0.17
*Aedes atlanticus* [29]	0.36	0.42	0.15
*Aedes taeniorhynchus* [21], [31]	0.49	0.27	0.13
*Aedes albopictus* [33]	0.52	0.25	0.13
*Culex salinarius* [31]	0.54	0.24	0.13
*Culex pipiens* [32], [34], [35]	0.13	0.90	0.12
*Aedes vexans* [21], [29]	0.26	0.41	0.11
*Aedes aegypti* [34]	0.70	0.11	0.08
*Aedes cantator* [31]	0.71	0.11	0.07
*Mansonia dyari* [29]	0.17	0.40	0.07
*Culex erythrothorax* [32]	0.17	0.26	0.04
*Culex erraticus* [32]	0.15	0.28	0.04
*Culex nigripalpus* [21], [29], [32]	0.06	0.24	0.01
*Anopheles bradleyi-crucians* [31]	0.17	0.05	0.01
*Aedes infirmatus* [29]	0.29	0.00	<0.01
*Anopheles crucians* [29]	<0.01	<0.01	<0.01
*Culex quinquefasciatus* [32], [34]	<0.01	0.14	<0.01
*Aedes dorsalis* [30]	0.32	<0.01	<0.01

a Average rate of mosquitoes, regardless of infection status, containing virus in their legs.

b Average rate of refeeding mosquitoes with a disseminated infection that transmitted virus.

c Average rate of disseminated infection after ingesting RVFV multiplied by percentage of mosquitoes with a disseminated infection that transmitted virus by bite.

Additional data points were estimated that describe transmission rates for *Ae. dorsalis* (Meigen), *Cx. erythrothorax* (Dyar), *Cx. tarsalis*, and *Cx. erraticus* (Dyar-Knab) mosquitoes that developed a disseminated infection based on the estimated transmission rates of Turell et al. [32]. These data were standardized with the same methodology described above. Vector competence (C*_v_*) was calculated by multiplying the fraction of mosquitoes that develop a disseminated infection after feeding on a viremic host by the transmission rate of mosquitoes with disseminated infection based on estimated values for an exposure viremia of 10^7.5^ PFU/ml [36].

### Vertebrate host competence

When mosquitoes feed on an infected vertebrate a fraction of those mosquitoes will become infectious depending on the intensity of the vertebrate host's viremia and the mosquito's susceptibility to the virus [37]. Experimental infection studies that exposed vertebrate species to RVFV and monitored post-infection viremias were used to create a host competence index (C*_i_*). The vertebrate reservoir competence index represents the relative number of infectious mosquitoes that may result from feeding on infected vertebrate hosts and is calculated as the product of susceptibility to infection, mean daily infectiousness to each species of mosquito, and duration of infectiousness [38]. Published studies were located using Web of Science, NCBI's Pubmed, and the Armed Forces Pest Management Board Literature Retrieval Systems. Studies utilizing PFU/ml and Tissue Culture Infectious Dose 50% (TCID_50_) techniques to quantify viral titers after experimental infection with virulent strains of RVFV (ZH501,T1,T46, AN1830, Kabete, 80612A, AnD100286, AnD100287, Z8548, FRhL2) were the only inclusion criteria for host competence data as no universal conversion between Lethal Dose 50% (LD_50_) and Mouse Lethal Dose 50% (MLD_50_) was found. Conversion from TCID_50_ to PFU/ml was obtained by the equation: PFU/ml = TCID_50_/ml×0.69 [39], [40].

To calculate the vertebrate host competence index for RVFV, an equation describing vector competence was calculated utilizing available mosquito transmission experiments performed at 26°C as a linear function of log (host viremia). This viremia-vector competence equation (Figure S1, C) describes the fraction of mosquitoes that would become infected after feeding on a single viremic host indicating the infectiousness of a vertebrate [37], [38]. Because of limited species-specific experimental transmission data, the viremia-vector competence equation is based on the combined experimental transmission data of 17 mosquito species (See Figure S1). Mosquito species that demonstrated low overall vector competence in experimental transmission studies due to midgut escape barriers or salivary gland barriers or had a limited sample size as described above were not used to calculate the viremia-vector competence relationship [29]. The viremia-vector competence equation (vector competence = 0.062 (Log_10_ viremia) −0.276; R^2^ = 0.27; N = 27; P = <0.001) was used to calculate the daily infectiousness of vertebrate hosts by inserting daily vertebrate host viremia titers into the equation. When the equation calculated a vertebrate host's infectiousness to be negative the vertebrate host's daily infectiousness was set to zero [37]. These daily values were summed over the host's viremic period and used as the vertebrate species' competence index (*C*_i_). When multiple experimental studies existed for a particular vertebrate species or taxonomic group a mean *C_i_* was calculated [37], [38], [41].

### Vector amplification fraction

To determine the theoretical importance of a mosquito to RVFV transmission it is important to consider contact rates between vectors and vertebrate hosts. The amplification fraction estimates the number of infectious mosquitoes resulting from feeding on a particular host and can be utilized as an index to compare the relative role of various vectors in transmission. In the WNV system, the relative number of infectious (transmitting) mosquito vectors resulting from feeding on a vertebrate host was estimated by Kent et al. [42] utilizing the following equation: *F_i_* = B*_i_^2^* * C*_i_* where *F_i_* = the relative number of infectious mosquitoes resulting from feeding on each vertebrate species *i*, where B*_i_* = the proportion of blood meals from species *i* and C*_i_* = reservoir competence. This equation was modified from Kilpatrick et al. [43] which estimated the fraction of WNV-infectious mosquitoes, *F_i_*, resulting from feeding on each avian species, *i*, as the product of the relative abundance, the vertebrate reservoir competence index, C*_i_*, and the mosquito forage ratio. Kent et al. [42] found that the relative abundance of each avian species cancelled out when multiplied by the forage ratio, of which the denominator is relative abundance. *F_i_* as defined by Kilpatrick et al. [43] was therefore reduced to the product of C*_i_* and the proportion of blood meals from species *i*. Because the viremia-vector competence relationship used in this analysis is based on data from multiple mosquito species, Kent et al's [42] *F_i_* equation was modified to multiply by the mosquito's vector competence value (*C_v_*) to account for the differences observed in mosquito vector transmission competence across species. The modified equation is referred to as the vector amplification fraction (*F_vi_*) and provides a theoretical means to compare the role of various vector species in the transmission of RVFV.
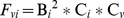
In the *F_vi_* equation, the number of infectious mosquitoes resulting from feeding on a vertebrate host, *F_vi_*, is equal to vertebrate host competence (*C*_i_), multiplied by the vector competence (*C_v_*), multiplied by the fraction of the total blood meals from host *i* squared (*B_i_* ^2^) [27], [42]. *B_i_* represents the number of blood meals taken from a vertebrate host species divided by the total blood meals taken. *B_i_* is unique to each mosquito species and is used as an indicator of exposure to RVFV and as an indicator of potential RVFV-infectious bites received by a host species, or taxonomic group [44]. Mosquito host feeding data from 39 studies were combined to generate a robust estimate of mosquito feeding patterns at the taxonomic resolution of Class and Order compiled into Table S2. Vertebrate hosts fed on by mosquitoes lacking a competence index (C*_i_*) were assigned the closest taxonomic mean [41]. Only mosquito species with over 40 recorded blood meals to calculate vertebrate host feeding proportions (*B_i_*) were included in this analysis. When vector competence data were missing for a given mosquito species, vector competence values were substituted based on the taxonomic subgenus average (*Aedes*- *Ochlerotatus*: 0.15; *Culex*- *Melanoconion*: 0.04, *Culex*: 0.11), genus average (*Anopheles*: <0.01; *Psorophora*: 0.18, *Mansonia*: 0.07) or family average (*Culicidae*: 0.15). To include *Ae. aegypti* in this analysis host-feeding patterns were estimated based on mosquito feeding patterns in Puerto Rico [45].

*F_vi_* is unique to each mosquito vector-vertebrate host pair and assumes initial seroprevalence, susceptibility and competence values are equal among all adult and juvenile vertebrate hosts [27], [46]–[47]. In an attempt to control any effect of the exposure dose of RVFV on the outcome of mosquito transmission competency, the *F_vi_* calculation only utilized mosquito competence values standardized to an exposure dose of 10^7.5^ PFU/ml as described above. To calculate a mosquito species' vector amplification fraction resulting from feeding on all vertebrate hosts, all *F_vi_* values reflecting a vector-vertebrate pair were summed for each mosquito species (equations shown in Table S3). This overall risk for a mosquito species to contribute to RVFV transmission in the U.S. was calculated based on a weighted percentage relative to the total *F_vi_* displayed by all mosquitoes.

### Vertebrate host amplification fraction

To explore the theoretical contribution of vertebrates to RVFV amplification and transmission in the U.S., *F_vi_* values unique to each vector-vertebrate pair described above were summed across each vertebrate host instead of by mosquito vector. The resulting index expresses the relative number of infectious mosquitoes generated by each vertebrate host. Since species-specific competence data was lacking for all vector-vertebrate host contacts, the role of vertebrate hosts was explored at the taxonomic resolution of class, order, and family. By summing *F_vi_* values with respect to vertebrate host at different taxonomic levels we were able to quantify the theoretical amplification fraction displayed by each vertebrate host taxonomic group. This index was expressed as a weighted average by dividing the summed *F_vi_* values for a vertebrate group by the total *F_vi_* value calculated for the mammalian order (Table S3).

## Results

### Vector competence

Eight experimental studies were identified that fit the inclusion criteria for this analysis [21], [29]–[35]. Data for 26 mosquito species were adjusted utilizing the viremic dose-dependent relationship of dissemination and transmission rates based on 17 species of mosquitoes (Figure S1, A and B). Standardized dissemination and transmission values were multiplied together to calculate vector competence (Table 1 and S1). The most competent transmission vectors of RVFV when exposed to 10^7.5^ PFU/ml of viremia are estimated to be *Coquillettidia perturbans* (Walker) (0.38), *Ae. japonicus japonicus* (Theobald) (0.37), *Cx. tarsalis* (0.33), and *Ae. excrucians* (0.28). Some mosquito species were estimated to be incompetent for RVFV, such as *An. crucians* (<0.01), *Ae. infirmatus* (<0.01), and *Cx. quinquefasciatus* (Say) (<0.01) (Table 1).

### Host competence

**Figure 1:**
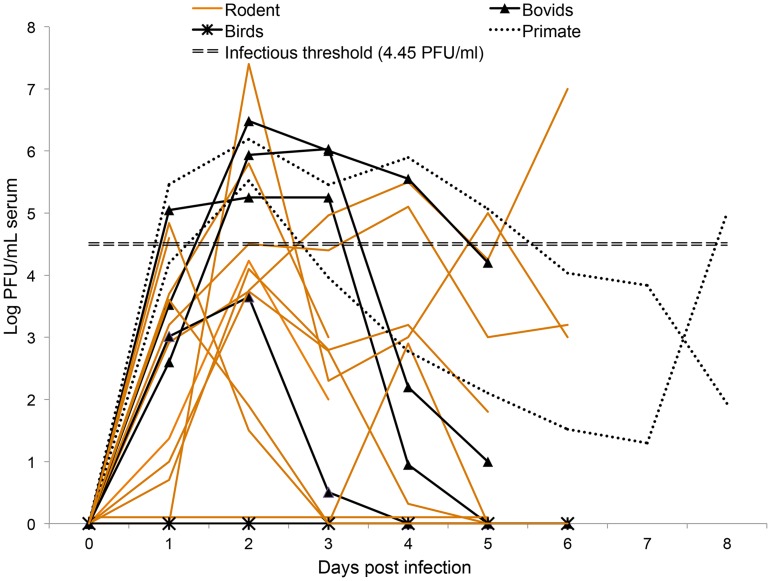
A graphical representation of the mean viremia profiles demonstrated by 20 different vertebrates after exposure to virulent strains of Rift Valley fever virus. Data was compiled from 17 published experimental infection studies and unpublished data from Dr. John Morrill and Dr. Michael Turell. Viral titers were quantified each day after infection by Plaque Assay or Tissue Culture Infectious Dose 50, which was converted to PFU/ml by the following equation: PFU/ml = TCID^50^/ml×0.69 [39], [40]. When a vertebrate host's viremia was calculated to be negative the daily infectiousness was set to zero as discussed in the methodology. References: Bovids: [48]–[51]; Birds: [52] (Turell unpublished data); Primate: [53]–[55] (Morrill unpublished data); Rodent: [56]–[65].

**Figure 2:**
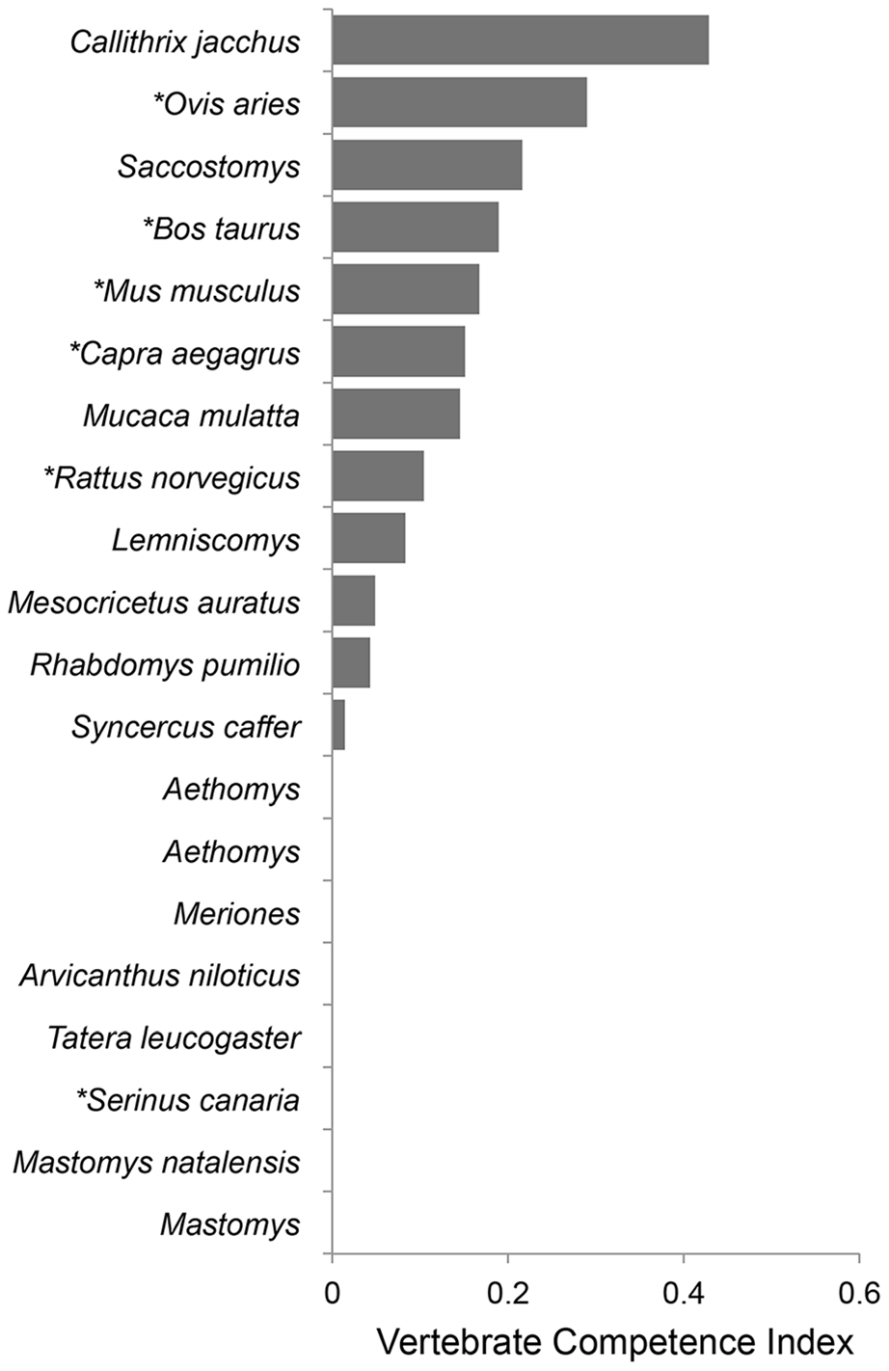
Rift Valley fever virus host competence index values for 20 vertebrate hosts based on experimental infection studies characterizing viremia profiles in PFU/ml or TCID^50^. The vertebrate host competence index value depends on the viral titer circulating in the blood and the duration of the infectious viremia [38]. Each value represents the sum of daily probabilities that an infected vertebrate host will transmit RVFV to a biting mosquito. This value was obtained by inserting the recorded daily viremia of experimentally infected hosts into the viremia-vector competence equation [% infectious = 0.062 (Log_10_ viremia)−0.276 (R^2^ = 0.27; p<0.001; N = 27)] (Figure S1, C). When a vertebrate host's viremia was calculated to be negative the daily infectiousness was set to zero. Conversion from TCID^50^ to PFU/ml was obtained by the equation: PFU/ml = TCID^50^/ml×0.69 [39], [40]. *Denotes a vertebrate species found in the U.S.

To estimate vertebrate host competence, published data and unpublished data provided by Dr. John Morrill from RVFV experimental infections (Figure 1) [39], [40], [48]–[65] were inserted into a viremia-vector competence equation that describes the relative number of infectious mosquitoes resulting from feeding on a vertebrate host (Figure S1, C). Exposure viremia dosages ranged from 10^4.3–10.2^ PFU/ml at an incubation temperature of 26°C. With this approach, 12 vertebrate species demonstrated reservoir competence by producing sufficient viremia titers to infect mosquitoes after exposure to RVFV, all of which were mammals (Figure 2) [38]–[40]. Vertebrate host species demonstrating competence for viral amplification were the following: sheep (*Ovis aries*, Class Artiodactyla), domestic cow (*Bos taurus*, Artiodactyla), domestic goat (*Capra aegagrus hircus*, Artiodactyla), mouse (*Mus musculus*, Rodentia); brown rat (*Rattus norvegicus*, Rodentia), the common marmoset (*Callithrix jacchus*, Primates); four-striped grass mouse (*Rhabdomys pumilio*, Rodentia); South African pouched mouse (*Saccostomus campestris*, Rodentia); Rhesus macaque (*Macaca mulatta*, Primates); Griselda's striped grass mouse (*Lemniscomys griselda*, Rodentia); African buffalo (*Syncerus caffer*, Artiodactyla); and namaqua rock rat (*Aethomys namaquensis*, Rodentia). Many species were considered incompetent because they did not develop a sufficient viremia profile to infect mosquito vectors (≤10^4.7^ PFU/ml), such as the red rock rat (*Aethomys chrysophilus*, Rodentia), African grass rat (*Arvicanthis niloticus*, Rodentia), guniea multimammate mouse (*Mastomys erythroleucus*, Rodentia), natal multimammate mouse (*Mastomys natalensis*, Rodentia), Mongolian gerbil (*Meriones unguiculatus*, Rodentia), Atlantic canary (*Serinus canaria*, Passeriformes), domestic chickens (*Gallus gallus*, Galliformes) and the Bushveld gerbil (*Taera leucogaster*, Rodentia).

The vertebrate host competence index averages based on taxonomy were the following: Class: Mammalian (0.17), Aves (0.00); Order: Primates (0.25), Artiodactyla (0.21), Rodentia (0.05); Family: Bovidae (0.21), Muridae (0.05), Cricitidae (0.05); Genus: *Ovis* (0.29), *Bos* (0.19), *Capra* (0.15), *Rattus* (0.04).

### Vector amplification fraction

Among mosquito species evaluated, the vector amplification fraction (Σ*F_vi_*) ranged from 0 to 0.018 (Table 2). The resulting index was expressed as a weighted percentage relative to the total amplification fraction demonstrated by the 40 mosquito species included in this analysis, which ranged from 0% to 11.7% (Table 2; See Table S3 for calculations). This index estimates the relative probability that a mosquito will feed on an infectious vertebrate host, develop a disseminated infection into the salivary glands, and ultimately transmit RVFV to a vertebrate host during a subsequent blood-feeding event. Mosquito species with the highest amplification fractions were: *Ae. japonicus japonicus* (Theobald) (11.4%), *Ae. thibaulti* (Dyar and Knab) (8.8%), *Ae. canadensis* (Theobald) (7.4%), *Culiseta inornata* (Williston) (6.7%), *Wyeomyia mitchellii* (Theobald) (6.6%), *Ae. sollicitans* (Walker) (5.4%), *Cq. perturbans* (5.4%), *Ae. sticticus* (Meigen) (5.4%), *Ae. aegypti* (5.0%) and *Ae. nigromaculis* (Ludlow) (4.4%) (Table 2).

**Table 2:** Relative risk of mosquitoes contributing to Rift Valley fever enzootic transmission in the U.S.

Mosquito Species	Vector Competence (C_v_)^a^	(ΣF_vi_)^b^	% Risk^c^
*Aedes japonicus japonicus*	0.37	3.10E-02	11.42%
*Aedes thibaulti*	0.15‡	2.30E-02	8.80%
*Aedes canadensis*	0.28	2.00E-02	7.42%
*Culiseta inornata*	0.15ƒ	1.80E-02	6.75%
*Wyeomyia mitchellii*	0.15ƒ	1.80E-02	6.63%
*Aedes sollicitans*	0.25	1.50E-02	5.37%
*Coquillettidia perturbans*	0.38	1.50E-02	5.36%
*Aedes sticticus*	0.15‡	1.40E-02	5.40%
*Aedes aegypti*	*0.08*	1.30E-02	5.04%
*Aedes nigromaculis*	0.15‡	1.20E-02	4.46%
*Aedes cantator*	0.07	9.60E-03	3.34%
*Psorophora columbiae*	0.18†	8.70E-03	3.25%
*Aedes trivittatus*	0.15‡	8.30E-03	3.12%
*Aedes fulvus pallens*	0.15‡	8.10E-03	3.04%
*Aedes taeniorhynchus*	*0.13*	7.80E-03	2.92%
*Psorophora discolor*	*0.18* †	7.00E-03	2.64%
*Psorophora ferox*	0.18	6.60E-03	2.49%
*Aedes albopictus*	0.13	5.90E-03	2.22%
*Aedes atlanticus*	*0.15*	5.70E-03	2.10%
*Mansonia titillans*	0.07†	4.70E-03	1.78%
*Aedes triseriatus*	0.24	4.30E-03	1.57%
*Aedes vexans*	0.11	3.30E-03	1.26%
*Culex erythrothorax*	0.04	3.10E-03	1.02%
*Culex salinarius*	0.13	1.90E-03	0.71%
*Culex cedecei*	0.04‡	1.00E-03	0.37%
*Deinocerites cancer*	0.15ƒ	9.90E-04	0.37%
*Culex tarsalis*	0.33	5.90E-04	0.22%
*Culex erraticus*	0.04	5.30E-04	0.19%
*Culex stigmatosoma*	0.11‡	3.70E-04	0.14%
*Culex nigripalpus*	0.01	3.30E-04	0.09%
*Culex restuans*	0.11‡	2.30E-04	0.09%
Anopheles crucians	<0.01	2.30E-04	0.08%
*Anopheles quadrimaculatus*	<0.01†	2.10E-04	0.08%
*Anopheles punctipennis*	<0.01†	2.10E-04	0.08%
*Culex pipiens*	0.12	1.70E-04	0.07%
*Culex pilosus*	0.04‡	1.20E-04	0.05%
*Culiseta moristans*	0.15ƒ	1.10E-04	0.04%
*Aedes infirmatus*	0	8.28E-05	0.03%
*Culex territans*	0.17	4.80E-06	0.00%
*Culiseta melanura*	0.15ƒ	3.40E-06	0.00%
*Culex peccator*	0.04‡	2.10E-07	0.00%
*Aedes dorsalis*	0	0.00E+00	0.00%
*Culex quinquefasciatus*	0	0.00E+00	0.00%

a Estimated Transmission Rate (*C_v_*) (Values from Table 1).

b *(*Σ*F_vi_)* for each mosquito species where F_i_ = B_i_^2^*C_i_ * C_v_.

c Σ*F_vi_*÷total *F_vi_* demonstrated by all mosquitoes.

† Genus average (*Anopheles*: <0.01; *Psorophora*: 0.18; *Mansonia*: 0.07).

‡ Subgenus average (*Aedes*- *Ochlerotatus*: 0.15; *Culex*: *Melanoconion*: 0.04, *Culex*: 0.11).

ƒ Family average substituted (Culicidae: 0.15).

### Vertebrate host amplification fraction

Overall four classes (Mammalia, Aves, Amphibia, and Reptilia), eight mammalian orders (Artiodactyla, Carnivora, Chiroptera, Didelphimorpha, Lagomorpha, Perissodactyla, Primates, Rodentia), six families (Bovidae, Cervidae, Cricitidae, Muridae, Sciuridae, Suidae) and seven genera (*Bos, Capra, Dama, Homo, Odocoilius, Ovis, Rattus*) of vertebrates were included in the model. As indicated by vertebrate competence studies, only mammals are competent hosts and are estimated to contribute 100% of theoretical RVFV amplification in the U.S. The order Artiodactyla is estimated to contribute 64.3% of all theoretical mammalian RVFV amplification followed by the orders Lagomorpha (16.8%), Primates (6.8%), Carnivora (4.4%), Rodentia (0.8%), Perissodactyla (0.4%), Didelphimorpha (0.1%), and Chiroptera (0.0%) (Table S3). Because some blood meal data was only specific to the taxonomic resolution of Class there were undefined mammalian hosts that represent 6.3% of the risk, which means all % risk estimates are potentially underestimated (Table S3). Similarly, within the Artiodactyla order 10.5% risk is undefined, therefore, the family Cervidae accounts for at least 56% of the theoretical RVFV amplification contributed to Artiodactyla, while Bovidae contributes 34%, and Suidae contributes <1% (Table S3).

## Discussion

### Vector competence

Rift Valley fever virus has been isolated from at least 40 African mosquito species and currently 19 North American species have been shown to be competent laboratory vectors of RVFV, several of which are known vectors of enzootic viruses of large mammals (e.g., *Cx. tarsalis* and western equine encephalitis virus or *Ae. taeniorhynchus* (Wiedemann) and Venezuelan equine encephalitis). These data suggest that a suite of mosquito vectors could potentially transmit RVFV should the virus reach North America [21].

Overall, results from previous studies have indicated that vector competence for RVFV is variable between mosquito species and among different populations of the same mosquito species. These variations in vector competence within mosquito species could be due to differences in development temperatures, phenotype, or parasite interactions that facilitate or block viral transmission [25], [32], [66]–[68]. Viral infection, dissemination and transmission rates are also dependent on the titer of the viremic exposure [33]. Because mosquito control methods vary for different mosquito species, future RVFV transmission experiments are necessary to better understand variations in vector competence [32], [68].

### Vertebrate host competence

The vertebrate host competence index value depends on the viral titer circulating in the blood and the duration of this infectious viremia [38]. As the classic RVFV transmission paradigm would hypothesize, which implicates peri-domestic livestock as important amplification hosts, the calculated vertebrate host competence index shows sheep, domestic cow, domestic goat, and African buffalo may potentially contribute to RVFV amplification (Figure 2) [69]. Primates from the new world also demonstrate a high competence suggesting humans may play a role in RVFV transmission. In the 1977 Egyptian outbreak of RVFV, Meegan et al. [6] demonstrated that humans produce a viremia of 10 ^4.1^–10 ^8.6^ LD_50_, but how this relates to vertebrate competence values of new world monkeys remains unclear. The vertebrate competence index indicates rodents can be competent amplification hosts, but their role in viral amplification may be limited as mosquitoes rarely use them as blood meal hosts. The lack of RVFV competence for parakeets, canaries, and pigeons has been described, however our analysis of the class Aves was limited to a study evaluating the Atlantic canary (*S. canaria*) [52] and an unpublished study by Turell et al. evaluating domestic chickens (*G. gallus*), both of which have a competence index of zero.

It is apparent that RVFV viremia profiles vary between vertebrate hosts (Figure 1 and Figure 2). These variations emphasize the importance of characterizing RVFV viremia profiles of domestic and wild animals present in the U.S., especially since their immune systems may be more susceptible to a foreign virus. Experimental infection studies evaluating vertebrate species from the U.S. with larger sample sizes will manifest in more accurate competence values and provide a finer set of data to better implicate important vertebrate hosts for RVFV amplification should the pathogen emerge in the U.S.

### Vector amplification fraction

Previous experimental transmission studies conclude that *Cx. tarsalis* and *Ae. j. japonicus* are the most competent vectors with the highest risk to transmit RVFV should it arrive in the U.S.; however, vector competence does not directly imply a significant role in disease transmission [21], [30]–[33], [36], [68]. The vector amplification fraction provides a means to quantitatively compare theoretical risk of various mosquito species based on their potential to contribute to RVFV transmission in the U.S. Vector-host contact rates, as dictated by mosquito feeding patterns, is a key component to consider when evaluating the risk of a mosquito vector, as illustrated by the *Cx. tarsalis* mosquioto. *Cx. tarsalis* is one of the most competent vectors of RVFV in the U.S. (Table 1), which feeds mainly on avian hosts (Table S2), and therefore, is predicted to have a low amplification fraction in comparison to other vectors as seen in Table 2 (0.2% of total risk). Recent transmission experiments by Turell et al. [30] suggest that *Ae. j. japonicus* mosquitoes are the most competent vector of RVFV in the U.S. (previously *Cx. tarsalis*). The vector amplification fraction calculated in this study further implicates *Ae. j. japonicus* as a high risk vector with the potential to contribute to RVFV transmission in the U.S. (11.4%, Table 2). This invasive mosquito has a high vector competence (0.37, Table 1), feeds heavily on competent hosts (Artiodactyla 80% and Primates 16%, Table S1), and is found in all U.S. states east of the Mississippi river except for Florida and Louisiana [70]. Should RVFV spread to the U.S., *Ae. j. japonicus* populations should be carefully monitored for infection and potentially targeted for mosquito control [30].

*Ae. sticticus* and *Cs. inornata* both demonstrate varying degrees of transmission competency, but vector competence for these two species remains undetermined. In the study by Iranpour [68], RVFV was detected in the saliva of *Ae. sticticus* after experimental infection and *Cs. inornata* demonstrated both a high infection rate (100%; N = 5) and high dissemination rate after exposure to RVFV viremia between 10^7.9^ to 10^9.4^ PFU/ml (60%; N = 3). Considering both these species feed heavily on the order Artiodactyla (*Ae. sticticus* 94% and *Cs. inornata* 80%, Table S2) their role in RVFV transmission in the U.S. is uncertain and should be evaluated. *Ae. trivittatus* is another mammal-biting mosquito estimated to have a moderate role in transmission that occurs in large populations in the Eastern U.S. and is lacking experimental data.

Among the top 10 mosquito species theoretically contributing to RVFV transmission in the U.S., only five species (*Ae. j. japonicus*, *Ae. sollicitans, Ae. canadensis*, *Cq. perturbans* and *Ae. aegypti*) have data comprehensive enough for this analysis. This underscores the lack in data necessary to estimate the theoretical role of different mosquito vectors in RVFV transmission in the U.S. Of those ranking as high-risk for contributing to RVFV enzootic transmission, some are limited in geographic range within the U.S. (e.g. *Wy. mitchellii*) underscoring the importance for including spatial and temporal mosquito abundance data while evaluating local regions for RVFV transmission potential. These results indicate a gap in experimental transmission data and requisite further vector competence evaluations to properly evaluate the potential risk of mosquitoes contributing to RVFV transmission in the U.S. Future studies should pay particular emphasis on assessing and re-evaluating the regional transmission competence and population dynamics of *Ae. j. japonicus*, *Cs. inornata, Ae. sollicitans, Ae. sticticus* (only 13 individuals have been evaluated [70]), *Ae. nigromaculis* (all data from one study in 1988 [31]), and *Ae. trivittatus* because of their estimated risk and abundance in the Eastern U.S.

### Vertebrate host amplification fraction

Artiodactyla, Lagomorpha, Primates, and Carnivora are estimated to be theoretically involved in RVFV amplification in the U.S., while the Mammalian orders Perissodactyla, Didelphimorpha and Chiroptera are not (Table S3). The order Chiroptera may deserve further investigation as a potential reservoir host as RVFV has been isolated from several bat genera [71] and even though antibodies against RVFV have been detected in horses, the family Equidae has demonstrated low viremic titers [72], [73].

Our results suggest that Artiodactyla contributes 64.3% of the theoretical risk for RVFV transmission in the U.S., which supports the currently held paradigm that Artiodactyla are the most important vertebrate host for RVFV amplification and transmission. Research and control efforts should place a particular emphasis on the families Cervidae and Bovidae as they account for at least 56% and 34% of the total risk contributed by the order Artiodactyla, respectively (Table S3). Based on the 2012 Census of Agriculture (USDA National Agriculture Statistics Service) there are about 90 million cattle, 5 million sheep, 3 million goats, and 300,000 captive cervids. There are an estimated 25 million white-tailed deer (*Odocoileus virginianus*) in the U.S. [74]. Throughout the U.S. captive and wild ruminants are widely available and heavily utilized by mosquitoes (Table S2) emphasizing their potential role in RVFV transmission.

It is important to note that the role of the order Lagomorpha (17%) may be inflated by the vector amplification fraction because their estimated vertebrate competence was based on a mammalian average (0.17). No studies provide evidence supporting that Lagomorphs are capable of producing an infectious viremia, but little research has evaluated their role in RVFV ecology [52]. Similarly, vertebrate competence of the order Carnivora is lacking. Studies demonstrate susceptibility in cats, dogs, ferrets and serological studies demonstrate antibodies against RVFV in lions (*Panthera leo*) and the polecat (*Ictonyx striatus*) [72], [75]–[77]. Experimental evaluation within the Order Carnivora should focus on the competence of dogs, cats, and raccoons because mosquito host-feeding is mainly associated with these species (Table S2).

Arbovirus amplification in domestic and peridomestic animals and eventual spillover to humans is a well-documented phenomenon. However the permanent establishment of dengue and chikungunya viruses in urban, tropical environments demonstrates the ability for arboviruses to subsist through human reservoirs [2], especially important given the recent emergence of chikungunya in the Caribbean in 2013 [76]. The vertebrate amplification fraction estimates Primates will contribute about 7% of the theoretical RVFV amplification in the U.S. (Table S3). This estimate is based on the assumption that the human viremia profile is comparable to Rhesus macaques and common marmosets. Viremia data from new-world monkeys as a surrogate for human viremia may overstate the role of humans in RVFV transmission. In the 1977 Egyptian outbreak of RVFV, Meegan et al. [6] demonstrated that indeed humans produce a viremia of 10 ^4.1^–10 ^8.6^ LD_50_, however socio-economic factors in the U.S. may limit mosquito-human contact rates, and dampen any role in amplification of RVFV. As such, the role of humans as vertebrate hosts for RVFV amplification remains unknown.

Hypotheses implicating rodents as important hosts for RVFV amplification started when high death rates of *Arvicanthis abyssinicus* and *Rattus rattus* coincided with sheep deaths caused by RVFV in 1932 [72]. Experimental studies demonstrate rodents can be competent amplification hosts for RVFV (Figure 1 & 2) depending on the viremic dose, age, and species [72]. However, results from the vertebrate amplification fraction suggest members of the order Rodentia are at low risk for contributing to RVFV transmission because of infrequent contact with mosquitoes (Table S2).

### Limitations

Given the gaps in data preventing a complete analysis of the amplification fraction potentially produced by all mosquito and vertebrate hosts, we made several assumptions that limit the accuracy of these results. This analysis does not account for spatial or temporal variation in mosquito abundance or competence, both of which are known to be spatially heterogeneous and influence pathogen transmission dynamics [32], [77]. Many of the mosquito species and vertebrate hosts included in the analysis have no competence data and for these species we assigned taxonomic averages. It is important to note that taxonomic averages are not always appropriate and extrapolations based on taxonomic averages for both vectors and vertebrate hosts can lead to spurious results (e.g. disparate RVFV vector competence exists for several *Culex* spp.) [41]. By combining data on 39 studies reporting mosquito host-feeding patterns in different regions and landscapes across the U.S, we aim to incorporate a robust measure of vertebrate host utilization. However, the mosquito host-feeding patterns for several species are based on a single study, and given the importance of host availability [78], a single study might not be broadly representative of host feeding patterns. Despite these limitations, the results from this study highlight potentially important mosquito vectors and vertebrate hosts of RVFV that should be monitored in the event RVFV emerges in the U.S. Additionally, this study identifies knowledge gaps that can be filled by future experimental work on both vectors and vertebrate species.

### Conclusion

World-wide zoonotic disease emergence is an increasing phenomenon due to environmental changes, ecological disturbances, and globalization [79]. The U.S. has already been affected by the emergence of WNV, recently identified a new zoonotic disease (Heartland virus) [80], [81], and is threatened by the spread of chikungunya virus to the Caribbean [76]. During the initial epidemics of WNV in the U.S. in 2002 and 2003, many mosquito control programs did not have a strong focus on *Culex* spp. mosquitoes. As knowledge of the WNV transmission system increased, vector control has improved by targeting *Culex* species to reduce human exposure events. The delay of *Culex* spp. vector control might have allowed more human WNV disease and may have contributed to the rapid spread of the virus across the U.S. highlighting the importance of *a priori* response strategies for potential viral threats.

RVFV is of particular concern in the U.S. because it causes disease in humans and economically important animals alike. Even more, its emergence throughout Africa and the Arabian Peninsula make it a conceivable threat for future geographic expansion. We combined published data to provide an estimate of each vector and vertebrate taxon's contribution to RVFV amplification in the U.S. However, major gaps in knowledge exist preventing a comprehensive evaluation of potentially important vectors and vertebrate hosts to RVFV transmission in the U.S. Results, combined with information on abundance of vectors and vertebrate hosts, can provide guidance for proactive management programs and aid parameterization for further modeling efforts evaluating environmental receptivity of RVFV in the U.S. [22], [28]. Additionally, the framework of this analysis can also be applied to regions in Africa and the Arabian Peninsula with endemic RVFV transmission to help identify important vectors and vertebrate hosts for vector control and vaccination programs.

Future research efforts should focus on: 1) further evaluating the dose-dependent nature of RVFV vector competence in geographically widespread mosquitoes quantified as high risk: *Ae. j. japonicus, Ae. canadensis, Cs. inornata, Ae. sollicitans, Cq. perturbans, Ae. sticticus, Ae. nigromaculis, Ae. cantator and Ae. trivitattus* 2) characterizing local vector competence in high risk areas for RVFV introduction, and 3) evaluating the RVFV viremia profiles of vertebrates in the U.S. with particular emphasis on the orders Artiodactyla (Cervidae, Bovidae, Suidae), Lagomorpha, and Carnivora (domestic dog, domestic cat, raccoon), respectively.

## Supporting Information

Figure S1 Dose-dependent relationship between exposure viremia and dissemination rate (A), transmission rate (B), and vector competence (C) displayed by 17 mosquito species in seven experimental transmission experiments: *Ae. aegypti, Ae. albopictus, Ae. atlanticus, Ae. canadensis, Ae. cantator, Ae. sollicitans, Ae. taeniorhynchus, Ae. triseriatus, Ae. vexans, Cq. perturbans, Cx. erraticus, Cx pipiens, Cx. salinarius, Cx. tarsalis, Cx. territans, Ma. dyari*, and *Ps. ferox*. Studies are cited in main manuscript.
(TIF)

Table S1 To standardize Rift Valley fever virus experimental transmission data two equations referenced in row 60 that estimate the viremia dose dependence of dissemination rate and transmission rate (see Figure S1-A and Figure S1-B) were utilized to interpolate what the dissemination and transmission rates would be at the exposure viremia of 10^7.5^ PFU/ml. A species average was calculated (Columns H and K) and multiplied together to calculate the vector competence at the same exposure viremia (Column L).
(XLS)

Table S2 Number and percentage of mosquito blood meals grouped by vertebrate host class and selected orders. Data is based on 39 combined mosquito feeding studies across the United States.
(DOCX)

Table S3 Vector competence data, vertebrate competence data, and mosquito feeding patterns were combined to estimate the Rift Valley fever virus amplification fraction displayed by the vectors and vertebrates in the United States. In the *F_vi_* equation (*F_vi_* = *B_i_* ^2^ * C*_i_* * C*_v_*), the number of infectious mosquitoes resulting from feeding on a vertebrate host, Fvi, is equal to vertebrate host competence (C*_i_*: located in row 5), multiplied by the vector competence (C*_v_*: located in column C), multiplied by the fraction of the total blood meals from host i squared (B*_i_* ^2^: indicated in each cell as a number divided by total blood meals in column B). All *F_vi_* values reflecting a vector-vertebrate pair were summed for each mosquito species (Column AC) and summed for each vertebrate species (Row 49). To present these values as a % risk (Column AD) the values of the vector amplification fraction were weighted over the total amplification demonstrated by all vectors, then multiplied by 100. To express the vertebrate contribution to RVFV amplification as a % risk (Row 50), the amplification values at the taxonomic resolution of Family and Order were weighted over the total amplification estimated by all mammals (Cell: Y49), then multiplied by 100. Because some blood meal data was only specific to the Mammalian class, 6.3% of the estimated amplification fraction is undetermined at the resolution of Order. Therefore, all order % risk estimates are minimum estimates.
(XLS)

## References

[1] Hartley DM, Rinderknecht JL, Nipp TL, Clarke NP, Snowder GD (2011) National Center for Foreign Animal and Zoonotic Disease Defense Advisory Group. Potential effects of Rift Valley fever in the United States. Emerg Infect Dis 17: 8. 10.3201/eid1708.101088 PMC3381545 21801607

[2] Weaver SC, Reisen WK (2010) Present and future arboviral threats. Antiviral Res 85: 328–345. 19857523 10.1016/j.antiviral.2009.10.008 PMC2815176

[3] Meegan J, Bailey CL (1988) Rift Valley fever. The arboviruses: epidemiology and ecology. Boca Raton, FL: CRC Press.

[4] Mandell R, Flick (2010) Rift Valley fever virus: An unrecognized emerging threat? Hum Vaccin 6: 597–601. 20421731 10.4161/hv.6.7.11761

[5] Ikegami T, Makino S (2011) The pathogenesis of Rift Valley Fever. Viruses 3: 493–519. 21666766 10.3390/v3050493 PMC3111045

[6] Meegan JM (1979) The Rift Valley fever epizootic in Egypt 1977–1978 1. Description of the epizootic and virological studies. Trans R Soc Trop Med Hyg 73: 618–623. 10.1016/0035-9203(79)90004-x 538803

[7] Fagbo S (2002) The evolving transmission pattern of Rift Valley Fever in the Arabian Peninsula. Ann N Y Acad Sci 969: 201–204. 12381591 10.1111/j.1749-6632.2002.tb04378.x

[8] CDC (2000) Outbreak of Rift Valley Fever –Yemen, August. MMWR 49: 1065–1066. 11186611

[9] CDC (2000) Outbreak of Rift Valley Fever Virus — Saudi Arabia, August. MMWR 49: 905–908. 11043643

[10] CDC (2000) Update: outbreak of Rift Valley fever—Saudi Arabia, August. MMWR 49: 982–985. 11098861

[11] WHO (2007) Outbreaks of Rift Valley fever in Kenya, Somalia and United Republic of Tanzania, December 2006–April 2007. Global Alert and Response 17508438

[12] WHO (2007) RVF, United Republic of Tanzania. Wkly Epidemiol Rec 82: 117–124. 17407852

[13] Bouloy M, Flick R (2009) Reverse genetics technology for Rift Valley fever virus: Current and future applications for the development of therapeutics and vaccines. Antiviral Res 84: 101–118. 19682499 10.1016/j.antiviral.2009.08.002 PMC2801414

[14] WHO (2010) Rift Valley fever in South Africa- update. Global Alert and Response

[15] Hassan OA, Ahlm C, Sang RC, Evander M (2011) The 2007 Rift Valley fever outbreak in Sudan. PLoS Negl Trop Dis 5: e1229. 21980543 10.1371/journal.pntd.0001229 PMC3181235

[16] Weaver SC (2005) Host range, amplification and arboviral disease emergence. In: Peters CJ, Calisher CH, editors. Infectious Diseases from Nature: Mechanisms of Viral Emergence and Persistence. Vienna: Springer. pp. 33–44. 10.1007/3-211-29981-5_4 16358422

[17] Pepin M, Bouloy M, Bird BH, Kemp A, Paweska J (2010) Rift Valley fever virus(Bunyaviridae: Phlebovirus): an update on pathogenesis, molecular epidemiology, vectors, diagnostics and prevention. Vet Res 41: 61. 21188836 10.1051/vetres/2010033 PMC2896810

[18] Bird BH, Nichol ST (2012) Breaking the chain: Rift Valley fever virus control via livestock vaccination. Curr Opin Virol 2: 315–323. 22463980 10.1016/j.coviro.2012.02.017

[19] Bird BH, Ksiazek TG, Nichol ST, Maclachlan NJ (2009) Rift Valley fever virus. J Am Vet Med Assoc 234: 883–893. 19335238 10.2460/javma.234.7.883

[20] Rolin AI, Berrang-Ford L, Kulkarni MA (2013) The risk of Rift Valley fever virus introduction and establishment in the United States and European Union. Emerg Microbes Infect 2: e81. 26038446 10.1038/emi.2013.81 PMC3880870

[21] Turell MJ, Dohm DJ, Mores CN, Terracina L, Wallette DL, et al (2008) Potential for North American mosquitoes to transmit Rift Valley Fever Virus. J Am Mosq Control Assoc 24: 502–507. 19181056 10.2987/08-5791.1

[22] Kakani S, LaBeaud AD, King CH (2010) Planning for Rift Valley fever virus: use of geographical information systems to estimate the human health threat of white-tailed deer (*Odocoileus virginianus*)-related transmission. Geospat Health 5: 33–43. 21080319 10.4081/gh.2010.185 PMC3140430

[23] Medlock JM, Hansford KM, Schaffner F, Versteirt V, Hendrickx G, et al (2012) A review of the invasive mosquitoes in Europe: ecology, public health risks, and control options. Vector Borne Zoonotic Dis 12: 435–447. 22448724 10.1089/vbz.2011.0814 PMC3366101

[24] Rose RI (2001) Pesticides nd public health: Integrated methods of mosquito management. Emerging Infectious Diseases 7: 17–23. 11266290 10.3201/eid0701.010103 PMC2631680

[25] Kilpatrick AM, Kramer Laura, Campbell Scott, Alleyne EO, Dobson Andrew, et al (2005) West Nile Virus risk assessment and the bridge vector paradigm. Emerg Infect Dis 11: 425–429. 15757558 10.3201/eid1103.040364 PMC3298247

[26] Hamer GL, Kitron UD, Goldberg TL, Brawn JD, Loss SR, et al (2009) Host selection by *Culex pipiens* mosquitoes and West Nile Virus amplification. Am J Trop Med Hyg 80: 268–278. 19190226

[27] Hamer GL, Chaves L, Anderson T, Kitron UD, Brawn JD, et al (2011) Fine-scale variation in vector host use and force of infection drive localized patterns of West Nile Virus transmission. PLoS ONE 6: e23767. 21886821 10.1371/journal.pone.0023767 PMC3158794

[28] Barker CM, Niu T, Reisen WK, Hartley DM (2013) Data-Driven modeling to assess receptivity for Rift Valley fever virus. PLoS Negl Trop Dis 7: e2515. 24244769 10.1371/journal.pntd.0002515 PMC3828160

[29] Turell MJ, Britch SC, Aldridge RL, Kline DL, Boohene C, et al (2013) Potential for mosquitoes (Diptera: Culicidae) from Florida to transmit Rift Valley fever virus. J Med Entomol 50: 1111–1117. 24180117 10.1603/me13049

[30] Turell MJ, Byrd BD, Harrison BA (2013) Potential for populations of *Aedes j. japonicus* to transmit Rift Valley fever virus in the USA. J Am Mosq Control Assoc 29: 133–137. 23923327 10.2987/12-6316r.1

[31] Gargan TP 2nd, Clark GG, Dohm DJ, Turell MJ, Bailey CL (1988) Vector potential of selected North American mosquito species for Rift Valley fever virus. Am J Trop Med Hyg 38: 440–446. 2895591 10.4269/ajtmh.1988.38.440

[32] Turell MJ, Wilson WC, Bennett KE (2010) Potential for North American mosquitoes (Diptera: *Culicidae*) to transmit Rift Valley Fever Virus. J Med Entomol 47: 884–889. 20939385 10.1603/me10007

[33] Turell MJ, Batley CL, Beaman JR (1988) Vector competence of a Houston, Texas strain of *Aedes albopictus* for Rift Valley fever virus. Infection 4: 5–9. 3193106

[34] Turell MJ, Linthicum KJ, Patrican LA, Davies FG, Kairo A, et al (2008) Vector competence of selected African mosquito (Diptera : Culicidae) species for Rift Valley fever virus. J Med Entomol 45: 102–108. 18283949 10.1603/0022-2585(2008)45[102:vcosam]2.0.co;2

[35] Turell MJ, Presley SM, Gad AM, Cope SE, Dohm DJ, et al (1996) Vector competence of Egyptian mosquitoes for Rift Valley fever virus. Am J Trop Med Hyg 54: 136–139. 8619436 10.4269/ajtmh.1996.54.136

[36] Turell MJ, Lee JS, Richardson JH, Sang RC, Kioko EN, et al (2007) Vector competence of Kenyan *Culex zombaensis* and *Culex quinquefasciatus* mosquitoes for Rift Valley Fever Virus. J Am Mosq Control Assoc 23: 378–382. 18240513 10.2987/5645.1

[37] Kilpatrick AM, LaDeau S, Marra P (2007) Ecology of West Nile Virus transmission and its impact on birds in the western hemisphere. The Auk 124: 1121.

[38] Komar N, Langevin S, Hinten S, Nemeth N, Edwards E, et al (2003) Experimental infection of North American birds with the New York 1999 strain of West Nile Virus. Emerg Infect Dis 9: 311–322. 12643825 10.3201/eid0903.020628 PMC2958552

[39] O'Reilly DR, Miller LK, Luckow VA (1994) The baculovirus expression vectors: a laboratory manual. Oxford University Press.

[40] Mena J, RamÃrez O, Palomares L (2003) Titration of non-occluded baculovirus using a cell viability assay. BioTechniques 34: 260–264. 12613247 10.2144/03342bm05

[41] Perez-Ramirez E, Llorente F, Jimenez-Clavero MA (2014) Experimental infections of wild birds with West Nile virus. Viruses 6: 752–781. 24531334 10.3390/v6020752 PMC3939481

[42] Kent R, Lara, Juliusson M, Weissmann S, Evans N (2009) Komar (2009) Seasonal blood-feeding behavior of *Culex tarsalis* (Diptera: Culicidae) in Weld County, Colorado, 2007. J Med Entomol 46: 380–390. 19351092 10.1603/033.046.0226

[43] Kilpatrick AM, Daszak P, Jones MJ, Marra PP, Kramer LD (2006) Host heterogeneity dominates West Nile virus transmission. Proc Biol Sci 273: 2327–2333. 16928635 10.1098/rspb.2006.3575 PMC1636093

[44] Muñoz J, Ruiz S, Soriguer R, Alcaide M, Viana DS, et al (2012) Feeding patterns of potential West Nile virus vectors in South-West Spain. PloS one 7: e39549. 22745781 10.1371/journal.pone.0039549 PMC3382169

[45] Barrera R, Bingham AM, Hassan HK, Amador M, Mackay AJ, et al (2012) Vertebrate Hosts of *Aedes aegypti* and *Aedes mediovittatus* (Diptera: Culicidae) in Rural Puerto Rico. J Med Entomol 49: 917–921. 22897052 10.1603/me12046 PMC4627690

[46] Dye C, Hasibeder G (1986) Population dynamics of mosquito-borne disease: effects of flies which bite some people more frequently than others. Trans R Soc Trop Med Hyg 80: 69–77. 3727001 10.1016/0035-9203(86)90199-9

[47] Woolhouse MEJ, Dye C, Etard JF, Smith T, Charlwood JD, et al (1997) Heterogeneities in the transmission of infectious agents: Implications for the design of control programs. Proc Natl Acad Sci U S A 94: 338–342. 8990210 10.1073/pnas.94.1.338 PMC19338

[48] Davies FG, Karstad L (1981) Experimental infection of the African buffalo with the virus of Rift Valley fever. Trop Anim Health Prod 13: 185–188. 7344184 10.1007/BF02237921

[49] Morrill JC, Carpenter L, Taylor D, Ramsburg HH, Quance J, et al (1991) Further evaluation of a mutagen-attenuated Rift Valley fever vaccine in sheep. Vaccine 9: 35–41. 2008798 10.1016/0264-410x(91)90314-v

[50] Rippy MK, Topper MJ, Mebus CA, Morrill JC (1992) Rift Valley fever virus-induced encephalomyelitis and hepatitis in calves. Vet Pathol Online 29: 495–502. 10.1177/030098589202900602 1448895

[51] Nfon CK, Marszal P, Zhang S, Weingartl HM (2012) Innate immune response to Rift Valley fever virus in goats. PLoS Negl Trop Dis 6: e1623. 22545170 10.1371/journal.pntd.0001623 PMC3335883

[52] Findlay GM, Daubney R (1931) The virus of Rift Valley fever or enzootic hepatitis. Lancet 2: 1350–1351.

[53] Peters CJ, Jones D, Trotter R, Donaldson J, White J, et al (1988) Experimental Rift Valley fever in *rhesus macaques* . Arch Virol 99: 31–44. 3355374 10.1007/BF01311021

[54] Morrill JC, Knauert FK, Ksiazek TG, Meegan JM, Peters CJ (1989) Rift Valley fever infection of rhesus monkeys: implications for rapid diagnosis of human disease. Res Virol 140: 139–146. 2756241 10.1016/s0923-2516(89)80091-3

[55] Smith DR, Bird BH, Lewis B, Johnston SC, McCarthy S, et al (2012) Development of a novel nonhuman primate model for Rift Valley fever. J Virol 86: 2109–2120. 22156530 10.1128/JVI.06190-11 PMC3302397

[56] Swanepoel R, Blackburn N, Efstratiou S, Condy J (1978) Studies on Rift Valley fever in some African murids (Rodentia: Muridae). J Hyg (Lond) 80: 183–196. 632561 10.1017/s0022172400053535 PMC2130003

[57] Anderson GW, Slone TW, Peters CJ (1987) Pathogenesis of Rift Valley fever virus (RVFV) in inbred rats. Microb Pathog 2: 283–293. 3509859 10.1016/0882-4010(87)90126-4

[58] Anderson GW Jr, Slone TW Jr, Peters CJ (1988) The gerbil, *Meriones unguiculatus*, a model for Rift Valley fever viral encephalitis. Arch Virol 102: 187–196. 3060046 10.1007/BF01310824

[59] Rossi CA, Turell MJ (1988) Characterization of Attenuated Strains of Rift Valley Fever Virus. J Gen Virol 69: 817–823. 3356978 10.1099/0022-1317-69-4-817

[60] Anderson GW, Lee JO, Anderson AO, Powell N, Mangiafico JA, et al (1991) Efficacy of a Rift Valley fever virus vaccine against an aerosol infection in rats. Vaccine 9: 710–714. 1759489 10.1016/0264-410x(91)90285-e

[61] Anderson GW, Rosebrock JA, Johnson AJ, Jennings GB, Peters CJ (1991) Infection of inbred rat strains with Rift Valley fever virus development of a congenic resistant strain and observations on age depencence of resistance. Am J Trop Med Hyg 44: 475–480. 2063951 10.4269/ajtmh.1991.44.475

[62] Pretorius A, Oelofsen MJ, Smith MS, Van Der Ryst E (1997) Rift Valley fever virus: a seroepidemiologic study of small terrestrial vertebrates in South Africa. Am J Trop Med Hyg 57: 693–698. 9430529 10.4269/ajtmh.1997.57.693

[63] Gora D, Yaya T, Jocelyn T, Didier F, Maoulouth D, et al (2000) The potential role of rodents in the enzootic cycle of Rift Valley fever virus in Senegal. Microbes Infect 2: 343–346. 10817634 10.1016/s1286-4579(00)00334-8

[64] Smith DR, Steele KE, Shamblin J, Honko A, Johnson J, et al (2010) The pathogenesis of Rift Valley fever virus in the mouse model. Virology 407: 256–267. 20850165 10.1016/j.virol.2010.08.016

[65] Geffers R, Schughart K, Panthier JJ, Zaverucha do Valler T (2010) GSE18064: Comparison of MBT/Pas and BALB/cByJ MEFs response after infection with Rift Valley Fever virus. Gene Expression Omnibus

[66] Turell MJ (1993) Effect of environmental temperature on the vector competence of *Aedes taeniorhynchus* for Rift Valley fever and Venezuelan equine encephalitis viruses. Am J Trop Med Hyg 49: 672–676. 8279634 10.4269/ajtmh.1993.49.672

[67] Vaughan JA, Turell MJ (1996) Facilitation of Rift Valley fever virus transmission by *Plasmodium berghei* sporozoites in *Anopheles stephensi* mosquitoes. American Journal of Tropical Medicine and Hygiene 55: 407–409. 8916797 10.4269/ajtmh.1996.55.407

[68] Iranpour M, Turell MJ, Lindsay LR (2011) Potential for Canadian Mosquitoes to Transmit Rift Valley Fever Virus. J Am Mosq Control Assoc 27: 363–369. 22329267 10.2987/11-6169.1

[69] Pepin M, Bouloy B, Bird BH, Kemp A, Paweska JT (2010) Rift Valley fever virus (*Bunyaviridae: Phlebovirus*): an update on pathogenesis, molecular epidemiology, vectors, diagnostics and prevention. Veterinary Research 41: 61. 21188836 10.1051/vetres/2010033 PMC2896810

[70] Kaufman MG, Fonseca DM (2014) Invasion Biology of *Aedes japonicus japonicus* (Diptera: Culicidae). Annu Rev Entomol 59: 31–49. 24397520 10.1146/annurev-ento-011613-162012 PMC4106299

[71] Calisher CH, Childs JE, Field HE, Holmes KV, Schountz T (2006) Bats: important reservoir hosts of emerging viruses. Clin Microbiol Rev 19: 531–545. 16847084 10.1128/CMR.00017-06 PMC1539106

[72] Olive MM, Goodman SM, Reynes JM (2012) The role of wild mammals in the maintenance of Rift Valley fever virus. J Wildl Dis 48: 241. 22493102 10.7589/0090-3558-48.2.241

[73] Yedloutschnig RJ, Dardiri AH, Walker JS (1981) The response of ponies to inoculation with Rift Valley fever virus. Cont Epidem Biostatist 3: 68–71.

[74] Miller KV, Muller LI, Demarais S (2003) White-tailed deer (*Odocoileus virginianus*). In: Feldhamer GA, Thompson BC, Chapman JA, editors. Wild Mammals of North America. Baltimore, MD: The Johns Hopkins University Press. pp. 906–930.

[75] Gear J, De Meillon B, Le Roux AF, Kofsky R, Innes RR, et al (1955) Rift Valley fever in South Africa: A study of the 1953 outbreak in the Orange Free State, with special reference to the vectors and possible reservoir hosts S. Afr Med J 29: 514–518. 14386032

[76] CDC (2014) Chikungunya in the Caribbean. Traveler's Health. Center for Disease Control and Prevention Centers for Diseaes Control and Prevention, NCEZID, DGMQ.

[77] Darsie RF, Ward RA (2005) Identification and geographical distribution of the mosquitos of North America, north of Mexico. Gainesville: University Press of Florida. xiv, 383 p. p.

[78] Chaves LF, Harrington LC, Keogh CL, Nguyen AM, Kitron UD (2010) Blood feeding patterns of mosquitoes: random or structured? Front Zool 7: 3. 20205866 10.1186/1742-9994-7-3 PMC2826349

[79] Patz JA, Graczyk TK, Geller N, Vittor AY (2000) Effects of environmental change on emerging parasitic diseases. Int J Parasitol 30: 1395–1405. 11113264 10.1016/s0020-7519(00)00141-7

[80] Savage HM, Godsey MS Jr, Lambert A, Panella NA, Burkhalter KL, et al (2013) First detection of heartland virus (*Bunyaviridae*: *Phlebovirus*) from field collected arthropods. Am J Trop Med Hyg 89: 445–452. 23878186 10.4269/ajtmh.13-0209 PMC3771279

[81] McMullan LK, Folk SM, Kelly AJ, MacNeil A, Goldsmith CS, et al (2012) A new phlebovirus associated with severe febrile illness in Missouri. N Engl J Med 367: 834–841. 22931317 10.1056/NEJMoa1203378

