# Supplementary figures and images for: Predicting the Mosquito Species and Vertebrate Species Involved in the Theoretical Transmission of Rift Valley Fever Virus in the United States
Source: PLoS Negl Trop Dis. 2014 Sep 11;8(9):e3163. doi: 10.1371/journal.pntd.0003163 (PMC4161329; doi:10.1371/journal.pntd.0003163)

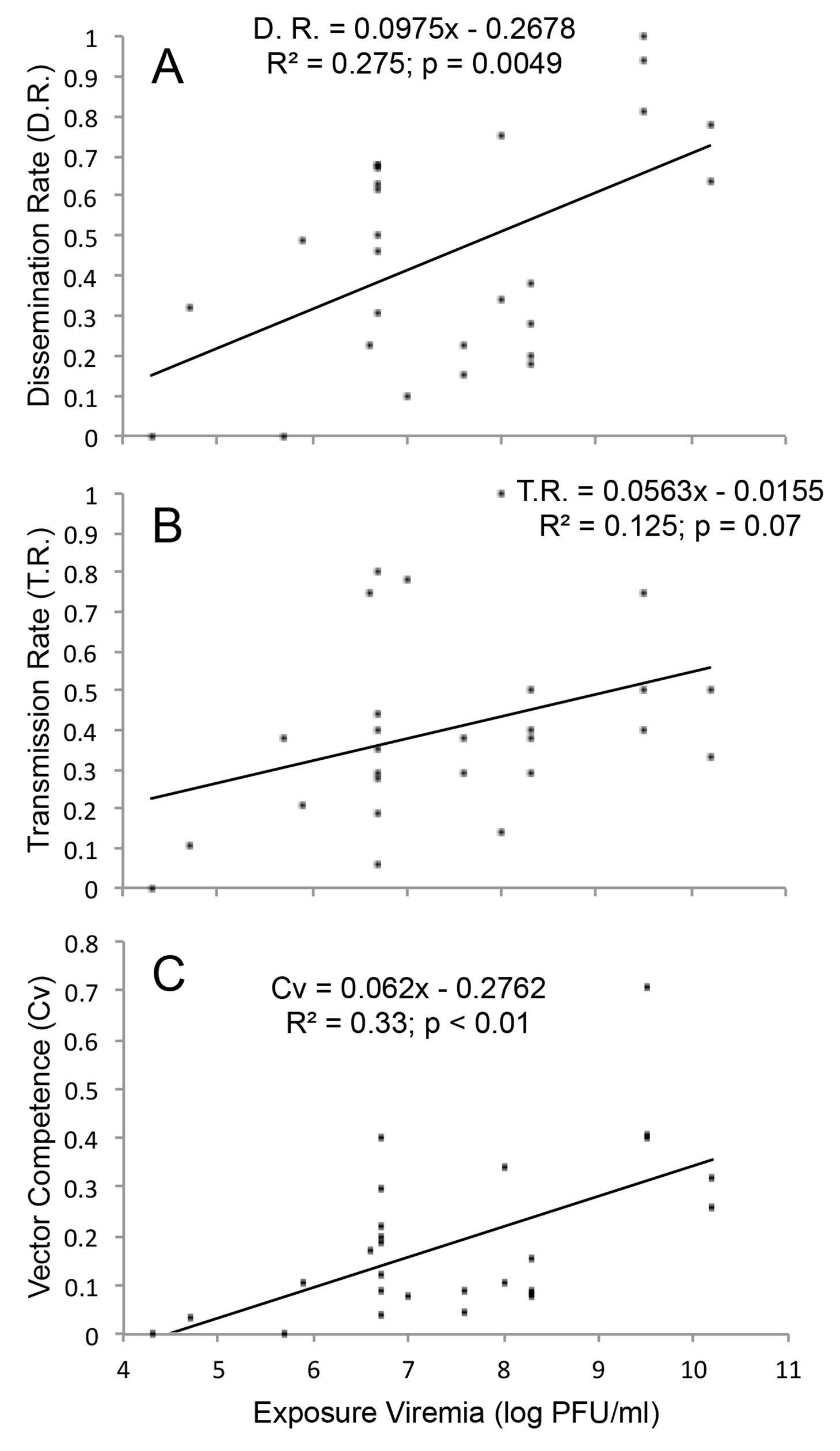

Supplement: Figure S1 — Dose-dependent relationship between exposure viremia and dissemination rate (A), transmission rate (B), and vector competence (C) displayed by 17 mosquito species in seven experimental transmission experiments: Ae. aegypti, Ae. albopictus, Ae. atlanticus, Ae. canadensis, Ae. cantator, Ae. sollicitans, Ae. taeniorhynchus, Ae. triseriatus, Ae. vexans, Cq. perturbans, Cx. erraticus, Cx pipiens, Cx. salinarius, Cx. tarsalis, Cx. territans, Ma. dyari, and Ps. ferox. Studies are cited in main manuscript. (TIF) [file pntd.0003163.s001.tif]
