# Supplementary material for: Predicting the Mosquito Species and Vertebrate Species Involved in the Theoretical Transmission of Rift Valley Fever Virus in the United States
Source: PLoS Negl Trop Dis. 2014 Sep 11;8(9):e3163. doi: 10.1371/journal.pntd.0003163 (PMC4161329; doi:10.1371/journal.pntd.0003163)
Supplement: Table S2 — Number and percentage of mosquito blood meals grouped by vertebrate host class and selected orders. Data is based on 39 combined mosquito feeding studies across the United States. (DOCX) [file pntd.0003163.s003.docx]

| Table S2. Number and percentage of mosquito blood meals by vertebrate host class and selected orders based on 39 combined mosquito feeding studies. | | | | | | | | | | | |
| --- | --- | --- | --- | --- | --- | --- | --- | --- | --- | --- | --- |
|  | Class | |  | Order | | | | | | |  |
| Taxon | Aves (%) | Mammalia (%) |  | Artiodactyla (%) | Carnivora (%) | Lagomorpha (%) | Perissodactyla (%) | Primates (%) | Rodentia (%) | Total | Citations |
| *Aedes aegypti* | 3(<1) | 439(99) |  | 0 | 81(18) | 0 | 1(<1) | 356 (80) | 0 | 442 | ^[1]^ |
| *Aedes* |  |  |  |  |  |  |  |  |  |  |  |
| *albopictus* | 1(<1) | 936(>99) |  | 26(3) | 381(41) | 92(10) | 45(5) | 223(24) | 106(11) | 937 | ^[2,3]^ |
| *Aedes atlanticus* | 1(1) | 123(92) |  | 34(26) | 6(5) | 41(31) | 1(1) | 1(1) | 1(1) | 133 | ^[4-6]^ |
| *Aedes* |  |  |  |  |  |  |  |  |  |  |  |
| *canadensis* | 2(1) | 242(73) |  | 202(61) | 3(<1) | 12(4) | 4(1) | 9(3) | 0 | 331 | ^[5,7,8]^ |
| *Aedes cantator* | 1(2) | 56(97) |  | 49(84) | 3(5) | 0 | 0 | 4(7) | 0 | 58 | ^[8]^ |
| *Aedes cinereus* | 5(5) | 105(95) |  | 80(73) | 6(5) | 2(2) | 1(<1) | 7(6) | 6(5) | 110 | ^[8]^ |
| *Aedes dorsalis* | 5(7) | 63(93) |  | 53(78) | 0 | 3(4) | 7(10) | 0 | 0 | 68 | ^[9,10]^ |
| *Aedes* |  |  |  |  |  |  |  |  |  |  |  |
| *f. pallens* | 2(5) | 39(95) |  | 4(10) | 7(17) | 6(15) | 0 | 0 | 1(2) | 41 | ^[4,5]^ |
| *Aedes infirmatus* | 29(3) | 828(97) |  | 103(12) | 14(2) | 340(40) | 0 | 0 | 1(<1) | 857 | ^[4,5]^ |
| *Aedes* |  |  |  |  |  |  |  |  |  |  |  |
| *j. japonicus* | 0 | 90(100) |  | 72(80) | 0 | 0 | 2(2) | 14(16) | 1(1) | 90 | ^[11,12]^ |
| *Aedes* |  |  |  |  |  |  |  |  |  |  |  |
| *nigromaculis* | 1(<1) | 297(<99) |  | 243(82) | 0 | 35(12) | 5(2) | 9(3) | 5(2) | 298 | ^[10]^ |
| *Aedes sollicitans* | 6(3) | 210(97) |  | 78(36) | 7(3) | 115(53) | 3(1) | 2(<1) | 2(<1) | 216 | ^[3,4,8,10,12,13]^ |
| *Aedes sticticus* | 0 | 79(100) |  | 74(94) | 1(1) | 2(3) | 1(1) | 1(1) | 0 | 79 | ^[8,9]^ |
| *Aedes* |  |  |  |  |  |  |  |  |  |  |  |
| *taeniorhynchus* | 17(3) | 644(97) |  | 265(40) | 30(5) | 285(43) | 2(<1) | 1(<1) | 3(<1) | 661 | ^[4,8,12]^ |
| *Aedes thibaulti* | 0 | 59(100) |  | 52(88) | 4(7) | 0 | 1(2) | 2(4) | 0 | 59 | ^[8]^ |
| *Aedes triseriatus* | 3(<1) | 484(89) |  | 53(10) | 92(17) | 57(10) | 14(3) | 31(6) | 223(41) | 543 | ^[4-6,8,12-14]^ |
| *Aedes trivittatus* | 8(3) | 253(97) |  | 119(46) | 76(29) | 30(11) | 11(4) | 15(6) | 0 | 261 | ^[8,10,12,14,15]^ |
| *Aedes vexans* | 47(<1) | 5351(99) |  | 3646(68) | 136(3) | 409(8) | 719(13) | 266(5) | 41(<1) | 5400 | ^[3,5,6,10,12-16]^ |
| *Anopheles* |  |  |  |  |  |  |  |  |  |  |  |
| *bradleyi* | 0 | 38(100) |  | 32(84) | 4(11) | 0 | 1(3) | 1(3) | 0 | 38 | ^[12]^ |
| *Anopheles* |  |  |  |  |  |  |  |  |  |  |  |
| *crucians* | 14(3) | 534(97) |  | 133(24) | 12(2) | 291(53) | 3(<1) | 1(<1) | 3(<1) | 548 | ^[4,5,12]^ |
| *Anopheles* |  |  |  |  |  |  |  |  |  |  |  |
| *punctipennis* | 3(3) | 85(97) |  | 52(59) | 4(5) | 1(1) | 25(32) | 3(3) | 0 | 88 | ^[5,7,8,10,12,17,18]^ |
| *Anopheles quad-* |  |  |  |  |  |  |  |  |  |  |  |
| *rimaculatus* | 23(2) | 940(98) |  | 476(49) | 271(28) | 23(2) | 125(13) | 17(2) | 1(<1) | 963 | ^[3-5,7,8,12,14,17,18]^ |
| *Coquillettidia* |  |  |  |  |  |  |  |  |  |  |  |
| *perturbans* | 75(9) | 747(91) |  | 348(42) | 50(6) | 100(12) | 35(4) | 4(<1) | 3(<1) | 823 | ^[3-5,7,8,12-14,16]^ |
| *Culex cedecei* | 6(3) | 156(89) |  | 13(7) | 7(4) | 47(27) | 0 | 0 | 37(21) | 176 | ^[19]^ |
| *Culex erraticus* | 740(55) | 582(43) |  | 77(6) | 354(26) | 89(7) | 13(1) | 4(<1) | 6(<1) | 1350 | ^[5,10,12,16-21]^ |
| *Culex* |  |  |  |  |  |  |  |  |  |  |  |
| *erythrothorax* | 41 (13) | 275(87) |  | 6(2) | 17(5) | 55(17) | 5(2) | 0 | 0 | 316 | ^[22]^ |
| *Culex nigripalpus* | 48 (36) | 83(62) |  | 23(17) | 46() | 0 | 11(8) | 4(3) | 1(<1) | 134 | ^[18,23]^ |
| *Culex peccator* | 10(5) | 1(<1) |  | 0 | 0 | 0 | 0 | 0 | 0 | 181 | ^[5,19-21]^ |
| *Culex pilosus* | 1(<1) | 29(23) |  | 3(2) | 6(5) | 6(5) | 0 | 0 | 0 | 125 | ^[19]^ |
| *Culex pipiens* | 1941(85) | 348(15) |  | 48 (2) | 80(4) | 7(<1) | 18(1) | 159(7) | 14(1) | 2291 | ^[3,6,7,9,10,12-14,17,24-27]^ |
| *Culex quinque-* |  |  |  |  |  |  |  |  |  |  |  |
| *fasciatus* | 846 (57) | 641(43) |  | 30(2) | 453(30) | 100(7) | 12(1) | 12(1) | 5(3) | 1489 | ^[2,5,17,22,23,28,29]^ |
| *Culex restuans* | 660(79) | 173 (21) |  | 8 (1) | 74(9) | 8 (1) | 39 (5) | 36(4) | 2(<1) | 833 | ^[3,5-7,10,13,14,17,24,26,30]^ |
| *Culex salinarius* | 89(29) | 213(70) |  | 117(38) | 54(18) | 21(7) | 6(2) | 5(2) | 3(1) | 304 | ^[3,5,10,12,13,26]^ |
| *Culex* |  |  |  |  |  |  |  |  |  |  |  |
| *stigmatosoma* | 47(84) | 9(16) |  | 1(2) | 0 | 8(14) | 0 | 0 | 0 | 56 | ^[22]^ |
| *Culex*  *tarsalis* | 10527 (86) | 1661 (14) |  | 1217 (10) | 62(<1) | 110 (1) | 158 (1) | 33 (<1) | 17 (<1) | 12188 | ^[10,22,31-36]^ |
| *Culex territans* | 13(5) | 7(3) |  | 2(<1) | 2(<1) | 1(<1) | 2(1) | 0 | 0 | 263 | ^[5,7,8,10,12,17,20]^ |
| *Culiseta inornata* | 68(4) | 1566(96) |  | 1311(80) | 4(<1) | 35(2) | 188(11) | 4(<1) | 1(<1) | 1635 | ^[9,10,14,37]^ |
| *Culiseta*  *melanura* | 1929(96) | 41(2) |  | 22(1) | 2(<1) | 1(<1) | 9(<1) | 2(<1) | 1(<1) | 1977 | ^[5,7,12,20,26,37]^ |
| *Culiseta* |  |  |  |  |  |  |  |  |  |  |  |
| *moristans* | 173(92) | 16(9) |  | 10(5) | 0 | 0 | 5(3) | 0 | 0 | 189 | ^[7,26]^ |
| *Deinocerites* |  |  |  |  |  |  |  |  |  |  |  |
| *cancer* | 448(75) | 138(23) |  | 0 | 9(2) | 116(20) | 0 | 0 | 0 | 594 | ^[38]^ |
| *Mansonia titillans* | 3(7) | 38(93) |  | 21(51) | 7(17) | 1(2) | 0 | 0 | 0 | 41 | ^[4,10]^ |
| *Psorophora* |  |  |  |  |  |  |  |  |  |  |  |
| *confinnis* | 5(3) | 175(97) |  | 108(60) | 20(11) | 15(8) | 7(4) | 13(7) | 12(7) | 180 | ^[4,10]^ |
| *Psorophora* |  |  |  |  |  |  |  |  |  |  |  |
| *discolor* | 8(10) | 72(90) |  | 48(60) | 10(13) | 5(6) | 2(3) | 7(9) | 0 | 80 | ^[10]^ |
| *Psorophora ferox* | 9(2) | 446(98) |  | 159(35) | 46(10) | 150(33) | 1(<1) | 8(2) | 2(<1) | 456 | ^[4-6,8]^ |
| *Wyeomyia* |  |  |  |  |  |  |  |  |  |  |  |
| *mitchellii* | 0 | 46(100) |  | 4(9) | 0 | 38(83) | 0 | 0 | 0 | 46 | ^[39]^ |

Literature Cited

1. Barrera R, Bingham AM, Hassan HK, Amador M, Mackay AJ, et al. (2012) Vertebrate Hosts of *Aedes aegypti* and *Aedes mediovittatus* (Diptera: *Culicidae*) in Rural Puerto Rico. J Med Entomol 49: 917-921.

2. Dennett JA, Bala A, Wuithiranyagool T, Randle Y, Sargent CB, et al. (2007) Associations between two mosquito populations and West Nile virus in Harris County, Texas, 2003–06. J Am Mosq Control Assoc 23: 264.

3. Gingrich JB, Williams GM (2005) Host-feeding patterns of suspected West Nile virus mosquito vectors in Delaware, 2001-2002. J Am Mosq Control Assoc 21: 194-200.

4. Edman JD (1971) Host-feeding patterns of Florida mosquitoes. I. *Aedes, Anopheles, Coquillettidia, Mansonia* and *Psorophora*. J Med Entomol 8: 687-695.

5. Irby WS, Apperson CS (1988) Hosts of mosquitoes in the coastal plain of North Carolina. J Med Entomol 25: 85-93.

6. Richards SL, Ponnusamy L, Unnasch TR, Hassan HK, Apperson CS (2006) Host-feeding patterns of *Aedes albopictus* (Diptera: Culicidae) in relation to availability of human and domestic animals in suburban landscapes of central North Carolina. J Med Entomol 43: 543-551.

7. Nasci RS, Edman JD (1981) Blood-feeding patterns of *Culiseta melanura* (Diptera, *Culicidae*) and associated sylvan mosquitoes in southeastern Massachusetts Eastern Equine Enchepalitis enzootic foci. J Med Entomol 18: 493-500.

8. Molaei G, Andreadis TG, Armstrong PM, Diuk-Wasser M (2008) Host-Feeding Patterns of Potential Mosquito Vectors in Connecticut, USA: Molecular Analysis of Bloodmeals from 23 Species of *Aedes, Anopheles, Culex, Coquillettidia, Psorophora,* and *Uranotaenia*. J Med Entomol 45: 1143-1151.

9. Tempelis CH, Francy DB, Hayes RO, Lofy MF (1967) Variations in feeding patterns of seven culicine mosquitoes on vertebrate hosts in Weld and Larimer Counties, Colorado. Am J Trop Med Hyg 16: 111-119.

10. Edman JD, Downe AER (1964) Host-blood sources and multiple-feeding habits of mosquitoes in Kansas. Mosquito News 24: 154-160.

11. Molaei G, Farajollahi A, Scott JJ, Gaugler R, Andreadis TG (2009) Human bloodfeeding by the recently introduced mosquito, *Aedes japonicus japonicus*, and public health implications. J Am Mosq Control Assoc 25: 210-214.

12. Apperson CS, Hassan HK, Harrison BA, Savage HM, Aspen SE, et al. (2004) Host feeding patterns of established and potential mosquito vectors of West Nile virus in the eastern United States. Vector Borne Zoonotic Dis 4: 71-82.

13. Apperson CS, Harrison BA, Unnasch TR, Hassan HK, Irby WS, et al. (2002) Host-feeding habits of *Culex* and other mosquitoes (Diptera: Culicidae) in the Borough of Queens in New York City, with characters and techniques for identification of *Culex* mosquitoes. J Med Entomol 39: 777-785.

14. Hamer GL, Kitron UD, Goldberg TL, Brawn JD, Loss SR, et al. (2009) Host selection by *Culex pipiens* mosquitoes and West Nile virus amplification. Am J Trop Med Hyg 80: 268-278.

15. Nasci RS (1984) Variations in the blood-feeding patterns of *Aedes vexans* and *Aedes trivittatus* (Diptera: *Culicidae*). J Med Entomol 21: 95-99.

16. Hassan HK, Cupp EW, Hill GE, Katholi CR, Klingler K, et al. (2003) Avian host preference by vectors of eastern equine encephalomyelitis virus. Am J Trop Med Hyg 69: 641-647.

17. Savage HM, Aggarwal D, Apperson CS, Katholi CR, Gordon E, et al. (2007) Host choice and West Nile virus infection rates in blood-fed mosquitoes, including members of the *Culex pipiens* complex, from Memphis and Shelby County, Tennessee, 2002-2003. Vector Borne Zoonotic Dis 7: 365-386.

18. Cohen SB, Lewoczko K, Huddleston DB, Moody E, Mukherjee S, et al. (2009) Host feeding patterns of potential vectors of eastern equine encephalitis virus at an epizootic focus in Tennessee. Am J Trop Med Hyg 81: 452-456.

19. Edman JD (1979) Host-feeding patterns of Florida mosquitoes (Diptera: Culicidae) VI. *Culex (Melanoconion)*. J Med Entomol 15: 521-525.

20. Burkett-Cadena ND, Graham SP, Hassan HK, Guyer C, Eubanks MD, et al. (2008) Blood feeding patterns of potential arbovirus vectors of the genus *culex* targeting ectothermic hosts. Am J Trop Med Hyg 79: 809-815.

21. Cupp EW, Zhang DH, Yue X, Cupp MS, Guyer C, et al. (2004) Identification of reptilian and amphibian blood meals from mosquitoes in an eastern equine encephalomyelitis virus focus in central Alabama. Am J Trop Med Hyg 71: 272-276.

22. Reisen WK, Milby MM, Presser SB, Hardy JL (1992) Ecology of mosquitoes and St. Louis encephalitis virus in the Los Angeles Basin of California, 1987-1990. J Med Entomol 29: 582-598.

23. Mackay AJ, Kramer WL, Meece JK, Brumfield RT, Foil LD (2010) Host feeding patterns of *Culex* mosquitoes (Diptera: Culicidae) in East Baton Rouge Parish, Louisiana. J Med Entomol 47: 238-248.

24. Hamer GL, Kitron UD, Brawn JD, Loss SR, Ruiz MO, et al. (2008) *Culex pipiens* (Diptera: Culicidae): a bridge vector of West Nile virus to humans. J Med Entomol 45: 125-128.

25. Kilpatrick AM, Kramer LD, Jones MJ, Marra PP, Daszak P (2006) West Nile virus epidemics in North America are driven by shifts in mosquito feeding behavior. PLoS Biology 4: 606-610.

26. Molaei G, Andreadis TG, Armstrong PM, Anderson JF, Vossbrinck CR (2006) Host feeding patterns of Culex mosquitoes and West Nile virus transmission, northeastern United States. Emerg Infect Dis 12: 468-474.

27. Patrican LA, Hackett LE, Briggs JE, McGowan JW, Unnasch TR, et al. (2007) Host-feeding patterns of *Culex* mosquitoes in relation to trap habitat. Emerg Infect Dis 13: 1921-1923.

28. Molaei G, Andreadis TG, Armstrong PM, Bueno R, Jr., Dennett JA, et al. (2007) Host feeding pattern of *Culex quinquefasciatus* (Diptera: Culicidae) and its role in transmission of West Nile virus in Harris County, Texas. Am J Trop Med Hyg 77: 73-81.

29. Molaei G, Cummings RF, Su TY, Armstrong PM, Williams GA, et al. (2010) Vector-host interactions governing epidemiology of West Nile Virus in Southern California. Am J Trop Med Hyg 83: 1269-1282.

30. Magnarelli LA (1977) Host feeding patterns of Connecticut mosquitos (Diptera-*Culicidae*). Am J Trop Med Hyg 26: 547-552.

31. Andersen DM, Collett GC, Winget RN (1967) Preliminary host preference studies of *Culex tarsalis* *Coquillett* and *Culiseta inornata* (Williston) in Utah Mosquito news 27: 12.

32. Gunstream SE, Chew RM, Hagstrum DW, Tempelis CH (1971) Feeding patterns of six species of mosqutoes in arid Southeastern California Mosquito News 31.

33. Kent R, Lara, Juliusson M, Weissmann S, Evans N, Komar (2009) Seasonal blood-feeding behavior of *Culex tarsalis* (Diptera: *Culicidae*)P in Weld County, Colorado, 2007. J Med Entomol 46: 380-390.

34. Lee JH, Hassan H, Hill G, Cupp EW, Higazi TB, et al. (2002) Identification of mosquito avian-derived blood meals by polymerase chain reaction-heteroduplex analysis. Am J Trop Med Hyg 66: 599-604.

35. Tempelis CH, Reeves WC, Bellamy RE, Lofy MF (1965) A three-year study of the feeding habits of *Culex tarsalis* in Kern County, California. Am J Trop Med Hyg 14: 170-177.

36. Wekesa JW, Yuval B, Washino RK, deVasquez AM (1997) Blood feeding patterns of *Anopheles freeborni* and *Culex tarsalis* (Diptera: *Culicidae*): effects of habitat and host abundance. Bulletin of Entomological Research 87: 633-641.

37. Edman JD, Kale HW, Webber LA (1972) Host-feeding patterns of Florida mosquitoes 2. *Culiseta*. J Med Entomol 9: 429-434.

38. Edman JD (1974) Host-feeding patterns of Florida mosquitoes. IV. *Deinocerites*. J Med Entomol 11: 105-107.

39. Edman JD, Haeger JS (1978) Host-feeding patterns of Florida Mosquitoes V-*Wyomyia*. J Med Entomol 14: 477-479.
